# Efficacy of 8 mg lidocaine and 2 mg cetylpyridinium chloride (CPC) fixed-combination lozenges on sore throat pain intensity compared with 1 mg lidocaine and 2 mg CPC fixed-combination lozenges in subjects with sore throat due to upper respiratory tract infection: a randomized double-blind parallel-group single-dose study

**DOI:** 10.1186/s13063-018-3077-6

**Published:** 2018-12-12

**Authors:** Frank Donath, Pascal Mallefet, Stephen Garreffa, Rowland Furcha

**Affiliations:** 1 SocraTec R&D GmbH, Mainzerhofplatz 14, 99084 Erfurt, Germany; 2 GSK Consumer Healthcare Company, Route de l’Etraz 2, 1260 Nyon, Switzerland; 3 Novartis Oncology, One Health Plaza, East Hanover, NJ 07936 USA

**Keywords:** Sore throat, Sore throat pain intensity, Lidocaine, Cetylpyridinium chloride

## Abstract

**Background:**

Lozenges containing lidocaine and cetylpyridinium chloride (CPC) are commonly used for the treatment of sore throat. The lidocaine acts locally to provide pain relief and the CPC has an antiseptic effect. Mebucaine CL, a well-established fixed-combination sore throat lozenge, contains 1 mg lidocaine and 2 mg CPC. Single-agent lozenges containing 8 mg lidocaine have also been demonstrated to be significantly superior to placebo in confirmatory pain intensity assessments. This study compared a new lozenge formulation, containing 8 mg lidocaine and 2 mg CPC, with the currently marketed lozenge for the treatment and relief of sore throat symptoms in subjects diagnosed with a sore throat due to an upper respiratory tract infection (URTI).

**Methods:**

In this double-blind parallel-group study, 250 adults with a sore throat due to an URTI were randomized to receive a single lozenge containing either 8 mg lidocaine + 2 mg CPC (*n* = 125) or 1 mg lidocaine + 2 mg CPC (*n* = 125). The primary efficacy endpoint of the study was the change in sore throat pain intensity (STPI) between baseline (immediately pre-treatment) and the 2-h post-dose assessment, measured on a 100 mm visual analog scale. STPI was measured at baseline and regular intervals up to 240 min after the lozenge was administered (evaluated in clinic). Any difficulty in swallowing and time to onset and duration of the analgesic effect were also assessed.

**Results:**

No increase in efficacy was demonstrated with the higher dose of lidocaine. The difference in the 2-h post-dose change in STPI was not statistically significant between the treatments. There was only one statistically significant difference between the treatments in all of the efficacy outcomes assessed: pain relief scores at 4 h post-dose were higher with 1 mg lidocaine + 2 mg CPC than with 8 mg lidocaine + 2 mg CPC (*P* = 0.0461). The most commonly reported adverse event (AE) was a headache; the only other AE experienced by more than one subject was throat irritation. No severe adverse events were reported during the assessment period.

**Conclusions:**

The modest difference in the pattern of effectiveness between the two treatments observed in this study does not support use of the 8 mg lidocaine + 2 mg CPC lozenge.

**Trial registration:**

ClinicalTrials.gov, NCT01265446. Registered on 20 December 2010.

## Background

Lidocaine and cetylpyridinium chloride (CPC) are compounds well-established for the treatment of sore throat and minor infections of the mouth and pharynx when formulated in a lozenge. These compounds act locally in the mouth and throat. Lidocaine is an amide anesthetic that reversibly blocks the voltage-sensitive sodium channels in the membrane of a nerve cell. This blockage prevents impulses from being conducted along the sensory nerves and results in the inhibition of pain. Lidocaine is used in oral preparations in different doses and formulations [1, 2]. Its efficacy and safety have been well established [2–7]. CPC is a quaternary ammonium antiseptic with an antimicrobial effect against gram-positive bacteria and, to a lesser degree, gram-negative bacteria [8]. It increases the permeability of microbial cell membranes to ions and metabolites, which induces a loss of cellular enzymatic and metabolic material, including deoxyribonucleic acid. In addition to its antibacterial activity, CPC also inhibits the growth of some fungi and viruses. The efficacy of CPC lozenges in the treatment of sore throat has been demonstrated [9] (data on file, GlaxoSmithKline Consumer Healthcare).

A currently marketed and well-established fixed-combination sore throat lozenge (Mebucaine CL containing lidocaine; Novartis Consumer Health, a GlaxoSmithKline Consumer Healthcare Company, Basel, Switzerland) contains 1 mg lidocaine and 2 mg CPC. Given that single-agent 8-mg lidocaine lozenges have been demonstrated to be significantly superior to placebo in confirmatory pain intensity assessments [2], a new lozenge formulation containing 8 mg lidocaine and 2 mg CPC has been developed. The objective of this study was to assess the efficacy and safety of the new fixed-dose combination lozenge compared with that of the marketed 1 mg lidocaine and 2 mg CPC fixed-combination lozenge for the treatment and relief of symptoms in subjects diagnosed with a sore throat due to upper respiratory tract infection (URTI).

## Methods

### Overall study design and plan

A randomized double-blind two-treatment-arm parallel-group study was conducted with 250 subjects (125 in each treatment arm) at a single center (SocraTec R&D) in Erfurt, Germany. Eligible subjects were randomized to receive treatment with a lozenge containing either 8 mg lidocaine + 2 mg CPC or 1 mg lidocaine + 2 mg CPC.

After the initial screening, a washout period of 30 min to a maximum of 8 h was observed to eliminate the effects of any prior treatment. The subjects then took the lozenge under supervision without water. The in-clinic evaluation period was 4 h post-treatment administration. Other topical and systemic oropharyngeal treatments were not allowed during the in-clinic evaluation period. The subject randomization list was prepared by Novartis. Subject enrollment and assignment were performed by a designated staff member at SocraTec R&D. Blinding was maintained until all subjects had completed the study. The first subject was screened in December 2010. The last subject completed the study in April 2011.

### Inclusion and exclusion criteria

#### Inclusion criteria

Subjects were considered eligible for the study if they met the following criteria: (i) male or female aged ≥18 years, (ii) a primary diagnosis of sore throat due to URTI, (iii) symptom onset ≤48 h prior to screening and ≤ 56 h prior to dosing, (iv) a tonsillopharyngitis assessment (TPA) score ≥5 on the expanded TPA scale [10–12], and (v) a sore throat pain intensity (STPI) score of at least 60 mm on the STPI scale, both at screening and immediately before dosing. The STPI scale is a visual analog scale (VAS) of 100 mm.

#### Exclusion criteria

Subjects for whom any of the following criteria were observed at screening or immediately prior to the time of dosing were excluded from the study: (i) use of other investigational drugs at the time of or prior to enrollment; (ii) history of hypersensitivity to any of the study drugs or the listed excipients; (iii) women of childbearing potential who were not using an acceptable method of contraception, and pregnant or lactating women; (iv) evidence of mouth breathing, severe coughing, or any disease that could compromise breathing; (v) overt oropharyngeal bacterial or fungal infection or lower respiratory tract infection; (vi) severe renal, liver, or cardiac impairment or severe lung disease; (vii) history of malignancy of any organ system other than localized basal cell carcinoma of the skin; (viii) prior to dosing of treatment analgesics, use of nonsteroidal anti-inflammatory drugs, antipyretics, antitussives, decongestant medications, medicated confectionary, throat lozenges, throat pastilles, sprays or products with demulcent properties, medication for sore throat containing a local anesthetic, or antibiotics; (ix) any other painful condition with an intensity that could distract the attention from sore throat pain; or (x) history of alcohol abuse or heavy smoking.

### Removal of patients from therapy or assessment

The following events could have led to withdrawal from the study: (i) an adverse event (AE), (ii) a major protocol violation that resulted in a significant risk to the subject’s safety, (iii) a fever that, according to the investigator, could have interfered with the ability of the subject to participate in the study, (iv) withdrawal of informed consent, or (v) administrative problems.

### Study endpoints and assessment methodology

The primary efficacy endpoint of the study was the change in STPI between baseline and 2-h post-dose measured on a 100 mm VAS. STPI was assessed at baseline, and then 15, 30, 60, 90, 120, 180, and 240 min after lozenge administration [2–5, 13]. Response to treatment was defined as a reduction in STPI from baseline of at least 50% at any time during the 4-h post-dose efficacy assessment period.

The secondary efficacy endpoints included: (i) difficulty in swallowing, (ii) time to onset and duration of analgesic effect, and (iii) global assessment of study medication. Swallowing difficulty was assessed with a 100 mm VAS at baseline, and then 15, 30, 60, 90, 120, 180, and 240 min after lozenge administration.

Sore throat pain relief was measured 15, 30, 60, 90, 120, 180, and 240 min after lozenge administration. The assessment was performed after swallowing using a five-point ordinal scale as follows: 0 = no improvement, 1 = slight improvement, 2 = moderate improvement, 3 = a lot of improvement, and 4 = complete improvement.

The time to first onset of pain relief was measured as the time between lozenge administration and the first onset of pain relief noticed by the subject. The duration of the analgesic effect was defined as the time from the first pain relief score ≥2 to the first pain relief score of 0 or 1.

Upon completion of the 4-h assessment, subjects were asked to rate the lozenges, taking into account all the benefits and any side effects attributed to the study medication. Answers were recorded on a scale from 0 to 4 (0 = unacceptable, 1 = poor, 2 = fair, 3 = good, and 4 = excellent).

Safety data collected included: (i) all treatment-emergent AEs with their severity and relationship to the study drug (as assessed by the investigator) and (ii) all serious AEs, regardless of the suspected causality, which occurred from when the subject gave informed consent until 30 days after participation in the study. The number and percentage of subjects presenting at least one AE was presented by primary system organ class and preferred term as defined in the Medical Dictionary for Regulatory Activities (MedDRA). General physical examinations and oropharyngeal assessments were conducted. Vital signs and results of pregnancy tests were recorded for each subject.

### Treatments administered

Eligible subjects were randomized in a 1:1 ratio to one of two treatment arms: 8 mg lidocaine + 2 mg CPC or 1 mg lidocaine + 2 mg CPC. Given that pain perception may vary between genders [14], separate randomization lists were generated for males and females.

The investigational drug (8 mg lidocaine + 2 mg CPC) and the comparator (1 mg lidocaine + 2 mg CPC) were identical in color, size, and shape: rectangular white lozenges. Both treatments were supplied in identical-looking 10-lozenge blister packs labeled with a unique randomization number. This number corresponded to a treatment arm according to the randomization list. Study staff dispensed a single dose to the subject at the study site. The subject was instructed not to swallow or chew the lozenge and to let it dissolve in the mouth.

Fluid intake was not allowed from 1 h before the dosing until the completion of the 2-h assessments. After that period, and only upon request, a volume of 120 mL of water at room temperature was provided to the subject. This maximum volume of water or a portion of it was consumed within 30 min following the 2-h assessments to avoid interfering with the 3-h assessments.

### Data collection

Designated investigator staff entered all data required by the protocol into case report forms. Field monitors reviewed the forms for completeness and accuracy and ensured that missing information and inaccuracies were addressed by site personnel.

### Statistical analysis

Approximately 125 subjects per group were planned to be randomized into the study. This proposed sample size was to provide a sufficient database for an effect size of 0.4. This sample size provided >85% power to detect a difference between the two groups. The statistical analysis was performed using SAS version 9.1 (SAS Institute, Cary, NC, USA). All statistical tests were two sided, with a probability of type I errors being 0.05. The changes in STPI and in difficulty swallowing were analyzed using an analysis of covariance with treatment and gender as factors. Response to treatment was analyzed using logistic regression with treatment and gender included as main effects. Both pain relief and response to treatment were analyzed using logistic regression. Time to first onset of pain relief was analyzed using a Cox proportional hazards model stratified by gender including treatment as a factor and baseline sore throat pain as a covariate.

## Results

### Demographics and other baseline characteristics

**Fig. 1:**
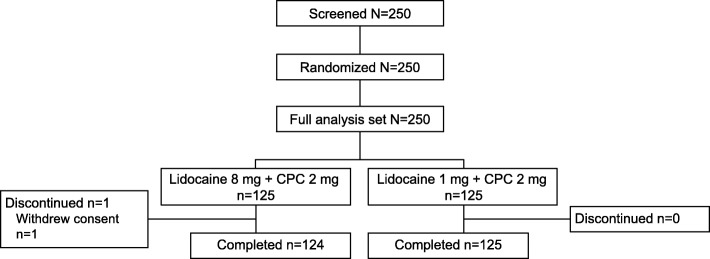
Study design. CPC cetylpyridinium chloride

A total of 250 subjects were included in the study, with 125 subjects in each group. One subject in the 8-mg lidocaine treatment arm withdrew consent after the 3-h efficacy assessment and did not complete the 4-h assessment (Fig. 1). Baseline demographic data and STPI scores are presented in Table 1. The distribution of characteristics measured was similar for both treatment arms.

**Table 1:** Baseline demographic data and STPI scores at screening and baseline (safety population)

	8 mg lidocaine + 2 mg CPC (*n* = 125)	1 mg lidocaine + 2 mg CPC (*n* = 125)	Total (*N* = 250)
Age, years	32.1 ± 12.6	31.6 ± 11.6	31.8 ± 12.1
Gender, *n* (%)			
Male	58 (46.4)	58 (46.4)	116 (46.4)
Race, *n* (%)			
Caucasian	124 (99.2)	124 (99.2)	248 (99.2)
Weight, kg	76.9 ± 19.6	78.6 ± 21.4	77.7 ± 20.5
Height, cm	173.2 ± 10.0	173.5 ± 9.8	173.3 ± 9.9
BMI, kg/m^2^	25.5 ± 5.6	25.9 ± 5.9	25.7 ± 5.7
STPI at screening, mm	71.5 ± 7.0	71.2 ± 6.7	71.4 ± 6.8
STPI at baseline, mm	72.3 ± 6.9	72.2 ± 6.8	72.2 ± 6.8

Data are expressed as mean ± standard deviation unless otherwise stated

*CPC* cetylpyridinium chloride, *BMI* body mass index, *STPI* sore throat pain intensity

### Treatment compliance

All 250 subjects randomized took one of the study medications. The mean time from the onset of the sore throat to study drug administration was 31.3 h in the 8-mg lidocaine and 31.6 h in the 1-mg lidocaine treatment arms. The maximum time from onset of the sore throat to study drug administration was 49.3 h, which was well within the limit of 56 h specified in the study protocol.

### Efficacy outcomes

#### Change in STPI

**Fig. 2:**
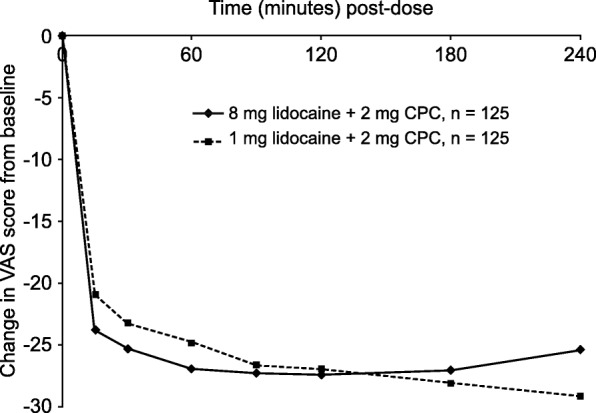
Change in sore throat pain intensity score from baseline. CPC cetylpyridinium chloride, VAS visual analog scale

Change in STPI score from baseline for the 4-h follow-up period is presented in Fig. 2. The mean change in STPI score from baseline to 2 h post-dose, the primary efficacy variable, was −27.4 mm in the 8-mg lidocaine treatment arm and −26.9 mm in the 1-mg lidocaine treatment arm. The median improvement was slightly greater in subjects given 8 mg lidocaine + 2 mg CPC (−26.0 mm) than in those given 1 mg lidocaine + 2 mg CPC (−23.0 mm). However, the difference between these treatments was not significant (*P* = 0.8597).

In the 8-mg lidocaine treatment arm, most of the reduction in STPI seen at any time point had already been achieved within 15 min of treatment. Mean change from baseline in VAS score was −23.7 mm at 15 min compared with −27.3 mm at 90 min, when maximum benefit was achieved. In the 1-mg lidocaine treatment arm, there was a steady improvement from 15 min to 4 h post-treatment. The mean reduction in STPI from baseline was −21.0 mm at 15 min. The STPI score continued to decrease and reached −29.1 mm at 4 h. The difference between treatments was not statistically significant at any time point.

#### Change in pain relief score

**Fig. 3:**
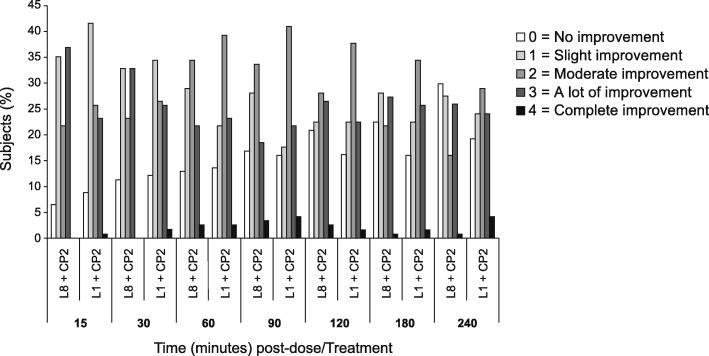
Sore throat pain relief scores. L1 + CP2 1 mg lidocaine + 2 mg cetylpyridinium chloride treatment arm, L8 + CP2 8 mg lidocaine + 2 mg cetylpyridinium chloride treatment arm

Figure 3 shows the sore throat pain relief scores for the 4-h post-dose evaluation period for the two treatment arms. At 15 min, when subjects treated with 8 mg lidocaine + 2 mg CPC experienced a greater reduction in STPI, 37% of subjects randomized to 8 mg lidocaine said that they had “a lot of” improvement, while only 24% of subjects randomized to the 1 mg lidocaine treatment arm said that they had “a lot of” or “complete” improvement. However, this difference was not statistically significant (*P* = 0.0527). At 30, 60, 90, 120, and 180 min, there was little difference in pain relief scores between treatments. Subjects treated with 1 mg lidocaine + 2 mg CPC reported a more long-lasting relief of sore throat pain. At 240 min, 57% of these subjects reported “moderate,” “a lot of,” or “complete” improvement, compared with 43% of subjects given 8 mg lidocaine + 2 mg CPC. This difference was statistically significant (*P* = 0.0461).

### Response to treatment

Altogether, 71 subjects (56.8%) randomized to 8 mg lidocaine + 2 mg CPC versus 69 subjects (55.2%) randomized with 1 mg lidocaine + 2 mg CPC responded to the treatment (*P* = 0.7820).

#### Time to first onset of pain relief, duration of analgesic effect, time to response to treatment

The median time to the first onset of pain relief, the median duration of the analgesic effect, and the median time to respond to treatment are presented in Table 2. Whereas the duration of the analgesic effect tended to be longer in subjects given the lower dose of lidocaine, the time to response to treatment tended to be shorter in those given the higher dose. However, these differences between treatments were not statistically significant.

**Table 2:** Time to first onset of pain relief, duration of analgesic effect, and time to response to treatment (full analysis set)

	8 mg lidocaine + 2 mg CPC (*n* = 125)	1 mg lidocaine + 2 mg CPC (*n* = 125)	*P*
Time to first onset of pain relief, median (95% CI) (min)	1.48 (1.35, 1.77)	1.57 (1.25, 1.75)	0.9471
Duration of analgesic effect, median (95% CI) (min)	165.0 (75.0, 224.0)	225.0 (163.0, −)	0.5365
Time to response to treatment, median (95% CI) (min)	120.0 (61.0, −)	180.0 (90.0, 244.0)	0.4875

*CPC* cetylpyridinium chloride, *CI* confidence interval

#### Change in difficulty swallowing

**Fig. 4:**
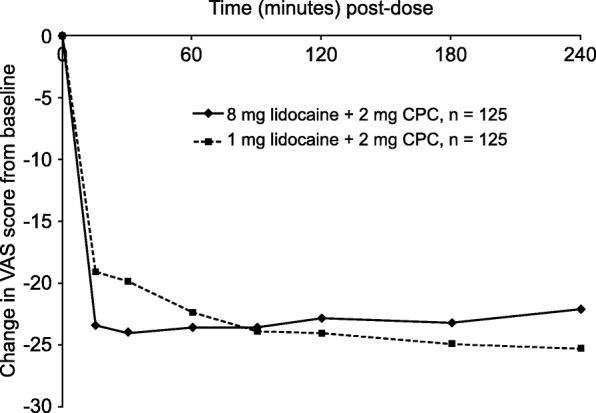
Change in difficulty swallowing from baseline for each post-dose time point. CPC cetylpyridinium chloride, VAS visual analog scale

A graphical overview of the changes in difficulty swallowing is presented in Fig. 4. For subjects treated with 8 mg lidocaine + 2 mg CPC, most of the improvement in swallowing was reached within the first 15 min post-treatment (−23.4 mm). The greatest difference was −23.9 mm, which was achieved after 30 min. The scores from subjects treated with 1 mg lidocaine + 2 mg CPC show a more gradual improvement in swallowing throughout the 240-min period, from −19.1 at 15 min to −25.3 at 240 min. A dose of 8 mg lidocaine + 2 mg CPC seemed more effective in improving swallowing within the first 60 min after treatment administration and 1 mg lidocaine + 2 mg CPC from 90 min onwards. These differences between treatments, however, did not reach statistical significance at any time point.

### Global assessment of study medication by treatment

**Fig. 5:**
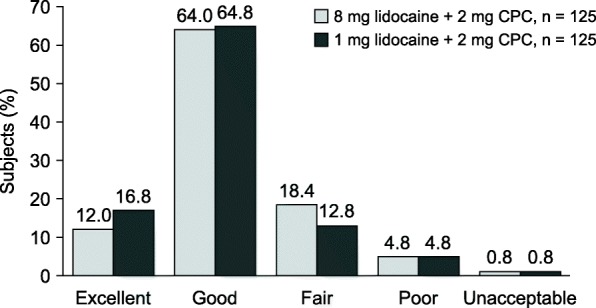
Global assessment of treatment after 4 h. Percentage of subjects in each treatment arm rating the treatment as excellent, good, fair, poor, or unacceptable. CPC cetylpyridinium chloride

Subjects were requested to rate their treatment at the end of the 4-h efficacy evaluation period. The results were very similar for both treatment arms (Fig. 5). Overall, 76% and 81.6% of subjects in the 8 mg lidocaine and 1 mg lidocaine treatment arms, respectively, evaluated their treatment as good or excellent. The treatment was found to be poor or unacceptable by 5.6% of subjects in each treatment arm.

### Safety

An overview of treatment-emergent AEs, regardless of their relationship with the study drug, by primary system organ class and preferred term is shown in Table 3. No severe AEs were reported during the assessment period. Two AEs of moderate severity were reported: hyperesthesia (8 mg lidocaine treatment arm) and headache (1 mg lidocaine treatment arm). The most commonly reported AE was headache, which could possibly be linked to the underlying URTI. The only other AE experienced by more than one subject was throat irritation.

**Table 3:** Number (*n*) and percentage (%) of treatment-emergent adverse events, regardless of study drug relationship, by primary system organ class, preferred term, and maximum severity^a^ (safety population)

System organ class and preferred term	8 mg lidocaine + 2 mg CPC (*n* = 125)		1 mg lidocaine + 2 mg CPC (*n* = 125)	
	Mild	Moderate	Mild	Moderate
Gastrointestinal disorders	1 (0.8)	0 (0.0)	1 (0.8)	0 (0.0)
Throat irritation	1 (0.8)	0 (0.0)	1 (0.8)	0 (0.0)
Respiratory, thoracic and mediastinal disorders	1 (0.8)	0 (0.0)	1 (0.8)	0 (0.0)
Asthma	0 (0.0)	0 (0.0)	1 (0.8)	0 (0.0)
Stridor	1 (0.8)	0 (0.0)	0 (0.0)	0 (0.0)
Nervous system disorders	8 (6.4)	1 (0.8)	6 (4.8)	1 (0.8)
Dizziness	0 (0.0)	0 (0.0)	1 (0.8)	0 (0.0)
Headache	7 (5.6)	0 (0.0)	5 (4.0)	1 (0.8)
Hyperesthesia	0 (0.0)	1 (0.8)	0 (0.0)	0 (0.0)
Vertigo	1 (0.8)	0 (0.0)	0 (0.0)	0 (0.0)
Skin and subcutaneous tissue disorders	1 (0.8)	0 (0.0)	0 (0.0)	0 (0.0)
Rash	1 (0.8)	0 (0.0)	0 (0.0)	0 (0.0)
Any primary system organ class	9 (7.2)	1 (0.8)	8 (6.4)	1 (0.8)

A subject with multiple occurrences of an AE under one treatment is counted only once in the AE category for that treatment. A subject with multiple AEs within a primary system organ class is counted only once in the total row

*CPC* cetylpyridinium chloride, *AE* adverse event

^a^No severe AEs were reported during the assessment period

Three subjects in the 8-mg lidocaine treatment arm and one subject in the 1-mg lidocaine treatment arm experienced AEs suspected by the investigator to have a causal relationship to study medication. These AEs were cases of throat irritation (one in each treatment arm), as well as cases of hyperesthesia, rash, and stridor (8-mg lidocaine treatment arm). The case of hyperesthesia was considered moderate in severity. All other AEs suspected to be related to the study drug were mild. None of the AEs led to withdrawal from the study. There were no deaths during the study.

One serious AE occurred within 30 days after treatment, in the 8-mg lidocaine treatment arm. Twenty-four days after treatment, the subject fell over, ruptured their Achilles tendon, and was hospitalized. This event was not suspected to have a causal relationship to the study medication.

There was no evidence of toxicity to any major organ system. Overall, both treatments were shown to be safe and generally well tolerated.

## Discussion

The present study was conducted and completed as planned. Of all subjects included, only one subject did not complete the study (withdrew consent). Protocol violations were minor, and demographic and baseline characteristics were similar across both study groups (8 mg lidocaine + 2 mg CPC and 1 mg lidocaine + 2 mg CPC).

The primary endpoint was not met. The difference in the change in STPI between baseline and the 2-h post-dose assessment was not statistically significantly different between treatments. There was only one statistically significant difference between treatments in all the efficacy outcomes assessed: pain relief scores at 4 h post-dose were higher with 1 mg lidocaine + 2 mg CPC than with 8 mg lidocaine + 2 mg CPC.

There was a difference in the pattern of effectiveness between the two treatments. Subjects treated with 8 mg lidocaine + 2 mg CPC experienced rapid pain relief. Within 15 min of treatment, they achieved a benefit close to the maximum benefit recorded in this treatment arm. However, this effect appeared not to be long-lasting. The median duration of the analgesic effect was approximately 3 h from dosing. Subjects treated with 1 mg lidocaine + 2 mg CPC had less relief of their symptoms in the first hour after dosing, but their symptoms then continued to improve throughout the entire 4-h efficacy assessment period. From 3 h post-dosing onwards, the lower dose of lidocaine tended to be more effective than the higher dose in providing pain relief.

The failure to demonstrate an increased efficacy with the higher dose of lidocaine was unexpected. Although a VAS has been used successfully to assess STPI in other studies, it has been used largely in placebo-controlled trials [2]. It is plausible that a VAS is not sensitive enough to discriminate between the subtle effects of the two highly effective concentrations of lidocaine studied here. Another possible explanation for this observation may relate to the origin of the sore throat in the subjects. In addition, it was reported anecdotally that the prohibition on drinking fluids for an hour before and for 2 h following dosing led to a build-up of phlegm. This may have led to discomfort, which could have masked the effect of the treatment.

## Conclusions

The 8 mg lidocaine + 2 mg CPC and 1 mg lidocaine + 2 mg CPC treatments were both shown to be safe and generally well tolerated. The primary endpoint was not met. Of all the efficacy outcomes assessed, only one statistically significant difference between treatments was observed. Pain relief scores 4 h post-dose were higher with 1 mg lidocaine + 2 mg CPC than with 8 mg lidocaine + 2 mg CPC. The modest difference in the pattern of effectiveness between the two treatments observed in this study does not support use of the 8 mg lidocaine + 2 mg CPC lozenge.

## Abbreviations

AE: Adverse event
BMI: Body mass index
CPC: Cetylpyridinium chloride
STPI: Sore throat pain intensity
URTI: Upper respiratory tract infection
VAS: Visual analog scale
